# Trans-cultural Adaptation and Validation of the “Teacher Job Satisfaction Scale” in Arabic Language Among Sports and Physical Education Teachers (“Teacher of Physical Education Job Satisfaction Inventory”—TPEJSI): Insights for Sports, Educational, and Occupational Psychology

**DOI:** 10.3389/fpsyg.2019.02234

**Published:** 2019-10-22

**Authors:** Nasr Chalghaf, Noomen Guelmami, Tania Simona Re, Juan José Maldonado Briegas, Sergio Garbarino, Fairouz Azaiez, Nicola L. Bragazzi

**Affiliations:** ^1^Department of Health Sciences (DISSAL), Postgraduate School of Public Health, University of Genoa, Genoa, Italy; ^2^Group for the Study of Development and Social Environment (GEDES), Faculty of Human and Social Science of Tunis, Tunis, Tunisia; ^3^Higher Institute of Sport and Physical Education of Sfax, University of Sfax, Sfax, Tunisia; ^4^Higher Institute of Sport and Physical Education of Kef, University of Jendouba, Jendouba, Tunisia; ^5^Research Unit, Sportive Performance and Physical Rehabilitation, High Institute of Sports and Physical Education of Kef, University of Jendouba, Jendouba, Tunisia; ^6^Department of Psychology and Sociology of Education, University of Extremadura, Badajoz, Spain; ^7^Department of Neuroscience, Rehabilitation, Ophthalmology, Genetics, Maternal and Child Health (DINOGMI), University of Genoa, Genoa, Italy

**Keywords:** trans-cultural adaptation and validation of a questionnaire, Arabic language, sports psychology, occupational psychology, job satisfaction, teachers

## Abstract

**Background:** Job satisfaction is largely associated with organizational aspects, including improved working environments, worker's well-being and more effective performance. There are many definitions regarding job satisfaction in the existing scholarly literature: it can be expressed as a positive emotional state, a positive impact of job-related experiences on individuals, and employees' perceptions regarding their jobs.

**Aims:** No reliable scales in Arabic language to assess job satisfaction in the sports and physical education field exist.This study aimed to trans-culturally adapt and validate the Pepe's “Teacher Job Satisfaction Scale” 9 items (TJSS-9), creating the “Teacher of Physical Education Job Satisfaction Inventory” (TPEJSI) in Arabic language. This scale was administered to a Tunisian population of sports and physical education teachers and analyzed according to the Pepe's theoretical model. More in detail, this investigation systematically tested its factor structure, in terms of internal consistency/reliability, predictive validity, sensitivity and convergent validity.

**Methods:** A total of 417 Tunisian teachers of sports and physical education participated voluntarily in this study. The sample comprised of 258 males and of 159 females. More in detail, 189 were teachers teaching in primary schools of physical education, 105 teaching in secondary schools, and 123 were university teachers. Both exploratory (EFA) and confirmatory (CFA) factor analyses were performed on random-split halves of the sample.

**Results:** The three-dimensional alpha coefficients of the TPEJSI were all >0.80: for satisfaction with colleagues, alpha was 0.865; for satisfaction with parents, alpha was 0.856 and for satisfaction with students alpha was 0.860. The CFA fit indices were satisfactory.

**Conclusions:** Given the good EFA factor loadings, the CFA fit indices, the correlation matrix, the sensitivity analysis, the convergent validity and the excellent internal consistency, it can be concluded that the TPEJSI is a good psychometric tool that can be used to quantitatively assess the job satisfaction level among teachers of sports and physical education in the Arabic-speaking world.

## Background

Job satisfaction is a multi-disciplinary concept that can be studied utilizing a variety of theoretical approaches and frameworks: it can be addressed from a psychological or sociological standpoint, as well as from the perspective of educational sciences and management (Hongying, 2007). As such, there exist different definitions of job satisfaction. According to Spector (1997), it represents a variable that measures the person's attitude to work, including the different facets of the job. Job satisfaction can be, as well, viewed as the result of the employee's interaction with and perceptions of his/her workplace and its surrounding environment (Carriere and Bourque, 2009; Rehman et al., 2013). Further, other researchers have defined job satisfaction as a positive emotional state (Inandi et al., 2013), a positive impact of the job-related experiences on individuals (Filiz, 2014), and employees' perceptions regarding their jobs and occupations (Altinkurt and Yilmaz, 2014).

In the literature, job satisfaction has been found to be inversely associated with absenteeism (Hanebuth, 2008), leaving the profession/retiring (MacIntosh and Doherty, 2010; Tschopp et al., 2014) and psychological stress (Moen et al., 2013).

By scanning the literature, it has been seen that there are very few studies investigating Arabic-speaking teachers' opinions about their job. In occupational psychology, having a reliable tool to measure the job satisfaction of employees is of crucial importance.

For this reason, in order to quantitatively evaluate job satisfaction among teachers, Pepe (2011) has developed the 9-item “Teachers Job Satisfaction Scale” (TJSS-9) as a psychometrically sound tool to be used in the academic field. More in detail, the TJSS-9 has three dimensions: namely, (i) the satisfaction of the colleagues (3 items), (ii) the satisfaction of the parents (3 items) and (iii) the satisfaction with the behaviors of the students (3 items). The current version of the instrument (9 items) was developed by Pepe (2011) from an original set of 35 elements covering six different dimensions: (i) satisfaction with all colleagues, (ii) satisfaction of colleagues, (iii) management satisfaction, (iv) satisfaction of parents, (v) satisfaction with students' behaviors, and (vi) responsibility.

## Aims

To the best of our knowledge and by scanning the existing scholarly literature, there is no psychometrically validated tool in Arabic language to assess the job satisfaction among sports and physical education teachers. Therefore, the major objective of the present study was to trans-culturally adapt and validate the TJSS-9 according to the Pepe's 3-dimensional theoretical model for sports and physical education teachers in Tunisia and to test its factor structure, in terms of internal consistency/reliability, predictive validity, and sensitivity. This study should be considered as a pilot, exploratory investigation, the results of which can pave the way for the development of further *ad hoc* instruments, specifically intended for sports and physical education teachers, correlating the job satisfaction level with other variables and constructs of interest.

## Materials and Methods

### Participants

A total of 417 sports and physical education teachers answered the questionnaire (159 and 258 males and females, respectively). Participants were divided according to their age into four categories: T1 (age <35; *n* = 113), T2 (35 ≤ age <40; *n* = 125), T3 (40 ≤ age <50; *n* = 100), and T4 (age ≥ 50; *n* = 79). Depending on their degrees, teachers are classified as physical education teachers (having completed a 2-year post-baccalaureate degree and having a 2-year university degree, *n* = 189), specialized secondary school teachers (having completed a 4-year training cycle after the baccalaureate and having a master degree, *n* = 105), and high sport institute teachers (*n* = 123). Teaching volumes varied according (22, 18, and 12 h *per* week, respectively).

### Ethical Approval

The study protocol of the present investigation received ethical clearance from the UNESCO Chair “Health Anthropology Biosphere and Healing Systems,” University of Genoa, Genoa (Italy), the Higher Institute of Sport and Physical Education of Sfax, Sfax (Tunisia), and the Higher Institute of Sport and Physical Education of Kef, Kef (Tunisia).

The project was approved by the Ethical Committee of the University of Sfax, Sfax, Tunisia.

All participants to the present study provided written, informed consent. Teachers were extensively informed about the purposes and procedures of the study, and were advised that the results would be made available to them upon completion of the study only in aggregate form, with no possibility to trace back to the single teacher's scores, thus ensuring anonymity and preserving the privacy of each participant.

The present investigation was carried out in accordance with the ethical principles of the 1964 Helsinki declaration and its subsequent amendments.

### Procedure

Teachers who agreed to participate in the study were instructed how to proceed and complete the survey procedures required by the present study. Following the agreement of the primary and secondary school principals, copies of the trans-culturally adapted TJSS-9 were distributed to teachers at their work sites in off-peak hours over a 2-month period, ensuring a proper duration (~30 min) in order to answer the questionnaire thoroughly.

### Statistical Analysis

#### Descriptive Analysis

Before commencing any statistical analysis (data handling, pre-processing, and analysis), data were visually inspected for potential outliers. Normality of data distribution was checked using the Kolmogorov-Smirnov test. Questionnaire scores were also checked for skewness and kurtosis, computing the Mardia's multivariate statistics.

For descriptive purposes, we calculated the mean and standard deviation (SD) of each score. The alpha level was set *a priori* at *P* ≤ 0.05.

#### Internal Consistency/Reliability

The internal consistency of the instrument was examined computing the Cronbach's alpha coefficient for all the 3 dimensions of the scale. More in detail, in order to properly interpret the alpha coefficient, the following rule of thumb was used (Nunnally, 1978): the coefficient was considered excellent if the estimate was >0.90, whereas it was deemed good in the range 0.80–0.90, acceptable in the range 0.70–0.80, questionable or adequate in the range 0.60–0.70, poor in the range 0.50–0.60 and unacceptable if <0.50.

#### Inferential Statistics—Sensitivity Analysis

The sensitivity of the instrument was tested by performing a multivariate analysis of variance (ANOVA), examining the impact of teachers' grade, gender, age, and their interaction effects on the TJSS-9 3 dimensions and total scores.

#### Exploratory Factor Analysis (EFA)

The factor structure was initially tested by carrying out an exploratory factor analysis (EFA) with the principal component analysis (PCA) and a *varimax* rotation with Kaiser Normalization. More in detail, *varimax* rotation was preferred to other kinds of rotation in that this approach, differently from the others, enables to minimize factor complexity while, at the same time, maximizing the variance of factor loadings (Tabachnick and Fidell, 2013).

Before proceeding with the EFA, the Kaiser-Meyer-Olkin (KMO) measure was computed in order to investigate the sampling adequacy. Ideal values of the KMO should be >0.60. Once verified the sampling adequacy, an EFA iterative strategy was implemented in the present study. More in detail, different PCA runs were conducted. First, an exploratory/preliminary PCA was conducted on the 9 items of the questionnaire without any rotation, in order: (i) to check if PCA could be judged an appropriate technique for the correlation matrix by assessing whether the correlations among items were satisfactory (that is to say, reporting values >0.30), and (ii) to control for the factorability of the correlation matrix computing the Bartlett's test of sphericity. In cases of statistical significance, this test enables to reject the null hypothesis that the correlations in the correlation matrix are zero and the matrix is an identity matrix.

The likely number of factors was found by: (i) calculating the number of factors with eigenvalues >1 (Field, 2009; Tabachnick and Fidell, 2013), and (ii) visually inspecting the Cattell's scree-plot. After checking the factor loadings, items were deleted in cases of unsatisfactory loading (that is to say, values <0.45). Moreover, items were not retained and suppressed if their factor loading conflicted with a sound theoretical explanation (Field, 2009; Tabachnick and Fidell, 2013).

Different PCAs with *varimax* rotation runs were, therefore, carried out in an iterative way, as previously explained, until a satisfactory, clearly interpretable solution was finally obtained. Cases of cross-loading were interpreted according to salience and explained variance, with theoretical considerations also being taken into account (Field, 2009; Tabachnick and Fidell, 2013).

#### Confirmatory Factor Analysis (CFA)

Then the model was tested by carrying out a confirmatory factor analysis (CFA). As suggested and recommended by many scholars (Hu and Bentler, 1995; Schumacker and Lomax, 2004), a wide range of fit indices was calculated and reported, namely: (i) discrepancy indices (including the chi-squared and the Steiger-Lind's Root Mean Square Error of Approximation or RMSEA), (ii) tests comparing the target model with the null model (like the Tucker-Lewis' Index or TLI, the Bentler's Comparative Fit Index or CFI, and the James-Mulaik-Brett's Parsimony Goodness-of-Fit Index or PGFI), and (iii) information theory goodness-of-fit measures (such as the Joreskog's goodness-of-fit index or GFI).

Regarding the cut-off and threshold values for discrepancy indices, the *p-value* associated with the chi-squared test should exceed 0.05. As far as the RMSEA is concerned, values higher than 0.10 indicate poor fitting models (Steiger, 2000). Concerning the cut-off and threshold values for tests that compare the target model with the null model, TLI should exceed 0.90 according to Byrne (1994) or 0.95 according to Schumacker and Lomax (2004) as well as according to Hu and Bentler (1995). PGFI is derived from NFI, correcting and compensating for model parsimony. CFI should exceed 0.95 (Bentler, 1990; Hu and Bentler, 1999) or 0.90 according to other scholars. Finally, regarding the cut-off and threshold values for information theory goodness-of-fit measures, GFI should be higher than 0.90 (Byrne, 1994).

### Convergent Validity

Convergent validity was measured by computing the Average Variance Extracted (AVE). More in detail, AVE measures the amount of variance that can be explained by the construct under study in relation to the amount of variance due to measurement error. Ideal values of AVE should be >0.5.

### Reliability

Reliability was assessed by calculating the composite reliability values, which ideally should be >0.7.

### Statistical Software

All statistical analyses were carried out using the commercial software “Statistical Package for the Social Sciences” (IBM SPSS software for Windows, version 21.0, IBM Corp., Armonk, NY, USA; released 2012) whereas the CFA was performed by utilizing the commercial software “Analysis of a moment structures” (Amos software for Windows, version 21.0, IBM, SPSS, Chicago, USA) (Arbuckle, 2012a,b).

EFA was conducted on a random-split half of the sample, whereas CFA was run on the other split half of the sample.

For all statistical analyses, figures with *p*-value less than 0.05 were considered statistically significant.

## Results

### Univariate and Multivariate Normality

Data were normally distributed in terms of skewness and kurtosis, whereas the multivariate Mardia test revealed violation of normality of distribution.

#### Adaptation of the Psychometric Instrument: The TPEJSI

The *ad hoc* devised psychometric instrument is made up of 9 items (3 items for each dimension), and the scores of the dimensions are obtained by averaging the items scores. The answers are coded on a 5-point Likert scale (see Table 1).

**Table 1:** Items of the *ad hoc* devised psychometric tool to quantitatively assess the job satisfaction level among the teachers of sports and physical education in the Arabic-speaking world, the “Teacher Physical Education Job Satisfaction Inventory” (TPEJSI).

**Teacher Physical Education Job Satisfaction Inventory (TPEJSI)** 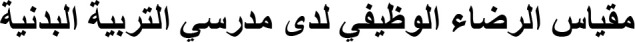	
**Items**	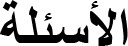
1. The quality of your relationships with your colleagues of sports and physical education at work	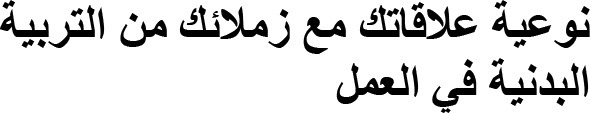
2. The extent to which your colleagues of sports and physical education encourage and support you in your work	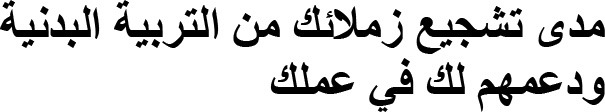
3. Your overall satisfaction with your colleagues of sports and physical education	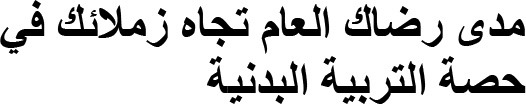
4. The extent of students' self-discipline behavior in the sports and physical education class	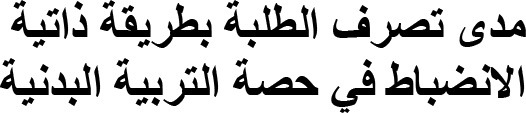
5. Your satisfaction with the behavior of students in the sports and physical education class	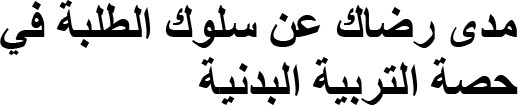
6. The overall level of satisfaction with students' discipline in sports and physical education class	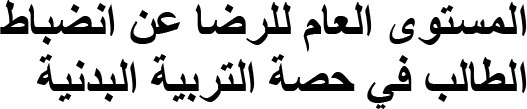
7. The degree of interest shown by parents toward their children being taught sports and physical education	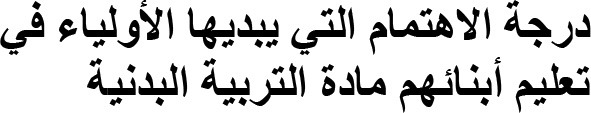
8. The extent to which parents support the school and its programs in sports and physical education	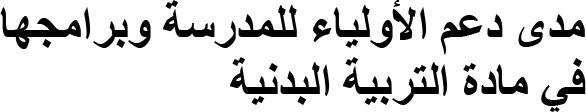
9. Your overall level of satisfaction with parents where you work	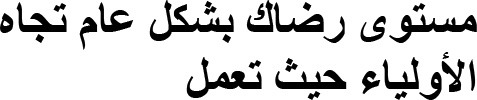

#### The Sensitivity Analysis

To test the sensitivity univariate analyses were conducted by gender, age, and grade for each dimension of the instrument. The descriptive statistics for the three dimensions are shown in Table 2 and the comparative statistics of the ANOVAs are shown in Table 3.

**Table 2:** Descriptive statistics reporting the scores of the “Teacher of Physical Education Burnout Inventory” (TPEJSI) for each dimension found performing the exploratory factor analysis (EFA). Scores are broken down by teachers' grade, gender, and age group.

**Gender**	**Age**	**Grade**	**CS**		**PS**		**SSB**	
			**Mean**	***SD***	**Mean**	***SD***	**Mean**	***SD***
Women	T1	TPS	3.03	0.25	3.32	0.17	3.68	0.18
		TSS	3.25	0.28	3.03	0.34	3.18	0.37
		UT	3.60	0.54	2.47	0.36	2.63	0.39
	T2	TPS	3.36	0.25	3.14	0.19	3.52	0.21
		TSS	3.26	0.40	3.25	0.26	3.47	0.28
		UT	2.82	0.33	2.53	0.22	2.78	0.24
	T3	TPS	2.82	0.27	2.82	0.22	2.99	0.24
		TSS	3.20	0.38	2.52	0.28	2.75	0.31
		UT	2.42	0.42	2.47	0.25	3.22	0.27
	T4	TPS	2.29	0.42	3.03	0.36	4.00	0.39
		TSS	2.90	0.38	2.88	0.34	3.64	0.37
		UT	2.00	0.36	2.33	0.21	2.53	0.23
Men	T1	TPS	3.40	0.18	3.52	0.24	3.22	0.26
		TSS	2.76	0.36	3.05	0.26	2.70	0.28
		UT	2.20	0.38	2.67	0.51	2.53	0.55
	T2	TPS	3.05	0.21	3.00	0.24	3.22	0.26
		TSS	2.98	0.28	3.26	0.38	3.07	0.41
		UT	3.09	0.23	2.67	0.32	2.90	0.34
	T3	TPS	3.14	0.24	2.80	0.25	3.13	0.27
		TSS	3.46	0.30	2.83	0.36	3.00	0.39
		UT	2.82	0.27	3.29	0.40	3.12	0.43
	T4	TPS	3.20	0.38	3.08	0.40	3.92	0.43
		TSS	2.58	0.36	3.07	0.36	3.17	0.39
		UT	2.23	0.22	2.12	0.34	2.79	0.37

*CS, colleague's satisfaction; PS, parent's satisfaction; SSB, satisfaction with student's behavior; SD, standard deviation; TPS, teacher of primary school; TSS, teacher of secondary school; UT, university teacher*.

**Table 3:** Univariate analyses for the three dimensions of the “Teacher of Physical Education Job Satisfaction Inventory” (TPEJSI).

	**CS**	**PS**	**SSB**
Gender	0.001	1.058	0.950
Age	3.298^*^	0.989	1.134
Grade	3.527^*^	6.026	7.792^**^
Gender X age	1.927	0.489	0.464
Gender X grade	1.714	0.246	0.421
Age X grade	0.574	1.493	1.782
Gender X age X grade	1.437	0.392	0.244

* Statistically significant at p < 0.05;

** *Statistically significant at p < 0.01*.

*CS, colleague's satisfaction; PS, parent's satisfaction; SSB, satisfaction with student's behavior*.

The analysis of the variance showed significant differences in the first dimension by age and grade. Also, a very significant difference by grade was found in the third dimension. However, no interactions effect between gender, age, and grade was shown for all the three dimensions.

#### Exploratory Factor Analysis

The KMO and Bartlett sphericity test provided a very significant chi-squared = 1773.817 value at *p* < 0.001, with a total explained variance of 78.41%. EFA made it possible to extract three factors that explained, respectively up of 26.31, 26.16, and 25.93% of the total variance. Table 4 shows the matrix of components after rotation.

**Table 4:** Factor loadings for the 3-factor solution of the “Teacher of Physical Education Burnout Inventory” (TPEJSI) questionnaire.

**Item**	**Components**		
	**CS**	**PS**	**SSB**
I1	0.880		
I2	0.898		
I3	0.884		
I4			0.875
I5			0.893
I6			0.875
I7		0.869	
I8		0.902	
I9		0.879	

*Exploratory factor analysis was performed with varimax rotation and Kaiser Normalization*.

*CS, colleague's satisfaction; PS, parent's satisfaction; SSB, satisfaction with student's behavior*.

#### Internal Consistency

The alpha coefficients of the three factors of the TPEJSI were all >0.80: for the dimension of satisfaction of the colleagues, alpha was 0.865; for the dimension of satisfaction of the parents, alpha was 0.856 and, finally, the dimension of satisfaction with behaviors of the students, alpha was 0.860.

#### Confirmatory Analysis of the Structure of the TPEJSI

We performed a confirmatory factor analysis (CFA) for the measuring instrument using the robust method of “Maximum Likelihood Estimate.”

The results of the CFA indices showed a factorial structure consistent with the theoretical model tested for the developed version of the instrument (see Table 5).

**Table 5:** Fit indices obtained from the confirmatory factor analysis (CFA) of the “Teacher of Physical Education Job Satisfaction Inventory” (TPEJSI).

**χ^2^**	**GFI**	**TLI**	**CFI**	**PGFI**	**RMSEA**
44.499	0.977	0.982	0.96	0.988	0.045

*CFI, Comparative Fit Index; GFI, goodness-of-fit index; PGFI, Parsimony Goodness-of-Fit Index; RMSEA, Root Mean Square Error of Approximation; TLI, Tucker-Lewis' Index*.

The robustness of an item is given by a high load factor. Comrey and Lee (2013) suggests that a factorial weight >0.71 is considered excellent, >0.63 is considered very good, >0.55 is considered acceptable and <0.45 is considered poor.

**Figure 1:**
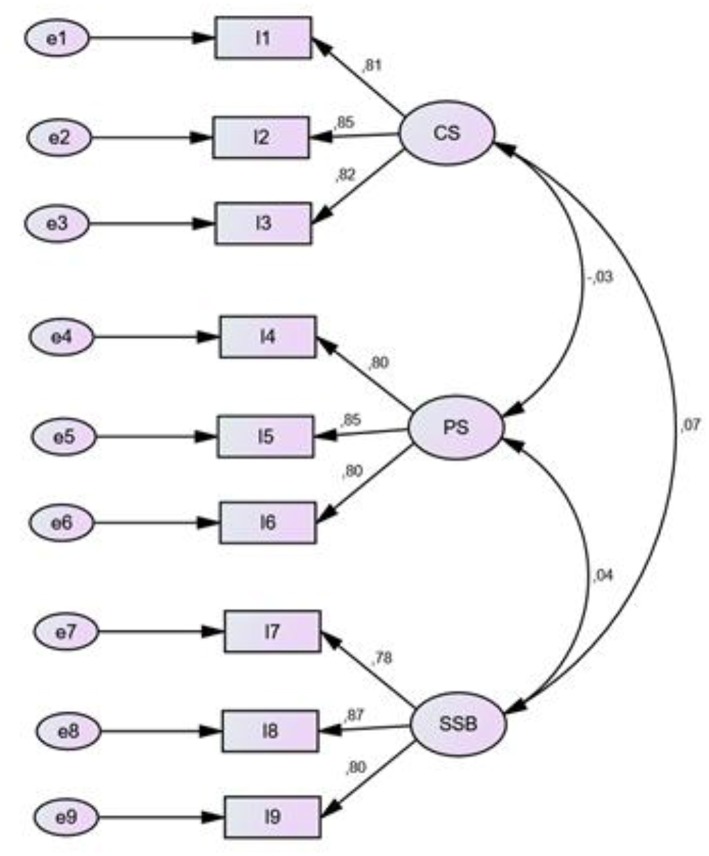
Findings of the confirmatory factor analysis (CFA) for the “Teacher of Physical Education Job Satisfaction Inventory” (TPEJSI). All parameters are significant at the 0.05 level.

In this study, CFA of the 9 items of TPEJSI showed excellent factorial weights (see Figure 1).

#### Convergent Validity of the Instrument

For the three dimensions, AVE values were 0.79, 0.78, and 0.78, respectively, indicating a good convergent validity of the instrument.

### Reliability of the Instrument

For the three dimensions, composite reliability coefficients were 0.92, 0.91, and 0.91, respectively, indicating a good reliability of the instrument.

## Discussion

The objective of the present study was to trans-culturally adapt, validate, and test the factor structure, internal consistency/reliability, predictive validity, sensitivity, and convergent validity of an *ad hoc* job satisfaction measurement scale for the Arabic-speaking world across sports and physical education teachers, devised according to the Pepe's 3-dimensional theoretical model. Once adapted, the 9-item tool was validated in a convenience sample of sports and physical education teachers in Tunisia, using both EFA and CFA. EFA factor loadings were good and the CFA fit indices satisfactory. The internal consistency/reliability of the 3 dimensions was found to be excellent.

To the best of our knowledge, no study has attempted so far to validate an adapted version of the TJSS-9 on a specific population of teachers in the Arabic-speaking world. The recent work of Pepe et al. (2017) has tested the invariance of the TJSS-9 in six countries: the Netherlands, the United States, Russia, China, Italy, and Palestine for a total of 2,819 active teachers. CFA and multi-group invariance tests were applied. TJSS-9 displayed robust psychometric properties and did not differ significantly between groups in terms of measurement invariance.

In agreement with our results, Pan et al. (2015) tested through a linear hierarchical regression model the relationship of job satisfaction with several demographic and work-related variables, such as gender, age, marital status, educational level, and job position. Results showed that only age and marital status were significant independent predictors of job satisfaction among Chinese university teachers. Furthermore, the effect of gender on the job satisfaction of British academics has been studied by Oshagbemi (2000), reporting that gender does not directly affect the job satisfaction of university teachers. However, the effect of gender interaction and grade was statistically significant (at *p* < 0.05). Overall, university women with higher grades tended to have higher job satisfaction scores. In another study by Wang and Lee (2009) conducted among Chinese university teachers, female teachers had slightly higher levels of satisfaction than men. Furthermore, teachers with the highest rank had the highest job satisfaction. Also, other scholars (De Nobile and McCormick, 2008; Demirtaş, 2010; Andersen, 2011; Magee, 2013) showed that gender can be either directly or indirectly associated with job satisfaction. While some investigations found that female teachers tended to exhibit a greater job satisfaction than their maile counterparts, other studies reported that gender was found to have a low level impact on teachers' job satisfaction. A recently published meta-analytical study conducted by Aydin et al. (2012) found an overall effect size of −0.02, in favor of male teachers.

Other studies carried out in Europe showed that female teachers were more satisfied in their job even though they were disadvantaged in terms of opportunities such us expectations about income, recruitment, and career advancement (Klassen and Chiu, 2010; Aydin et al., 2012; Saiti and Papadopoulos, 2015). Kaur and Sidana (2011) showed that the level of job satisfaction of male teachers was higher than their female counterparts.

Concerning the potential impact of the grade, many studies showed that job satisfaction levels presented significant differences between teachers teaching in primary, secondary or high schools. In this context, Demirtaş (2010) reported that the job satisfaction of teachers teaching in elementary schools was higher than the level of teachers teaching in secondary schools or in universities. Mhozya (2007) explored job satisfaction of primary school teachers in Botswana and found that a significant number of teachers were not satisfied with their salary with respect to the workload. Also, Indhumathi (2011) conducted a study among teachers of a secondary school, assessing the relationship between job satisfaction and performance. Author was able to prove, on the one hand, a significant association between job satisfaction and performance, and, on the other hand, significant differences in terms of job satisfaction across teachers based on their grade. On the contrary, Yazici and Altun (2013) could not find apparent differences among lecturers in universities in Turkey.

Furthermore, a considerable number of international studies have highlighted links between teachers' job satisfaction, workload and other related variables. For instance, Collie et al. (2012) reported that teacher job satisfaction was directly related to the perceived workload and the sense of effectiveness in teaching. In another study by Hoigaard et al. (2012), authors were able to replicate such findings, showed that teacher job satisfaction was positively related to teacher effectiveness and professional engagement.

However, the present study is not without limitations. The Arabic-speaking world is rather vast, heterogeneous and culturally different, therefore limiting the investigation to Tunisian subjects could influence the general extensibility of our results. As such, future studies from other Arabic-speaking countries are warranted to replicate our findings in a more statistically robust way.

## Conclusion

Our study aimed to trans-culturally adapt, validate and test the factor structure, internal consistency/reliability, predictive validity, sensitivity and convergent validity of a job satisfaction measurement inventory across Arabic-speaking sports and physical education teachers. Given the good EFA factor loadings, the CFA fit indices, the correlation matrix, the sensitivity analysis and the excellent internal consistency, it can be concluded that the TPEJSI is a good psychometric tool that can be used to quantitatively assess the job satisfaction level across teachers of physical education in the Arabic-speaking world. However, given the above-mentioned shortcomings, future studies in the field are urgently needed, also exploring the relationship of teacher job satisfaction with other psychological variables and constructs of interest.

## Data Availability Statement

The raw data supporting the conclusions of this manuscript will be made available by the authors, without undue reservation, to any qualified researcher.

## Ethics Statement

The study protocol of the present investigation received ethical clearance from the UNESCO Chair Health Anthropology Biosphere and Healing Systems, University of Genoa, Genoa (Italy), the Higher Institute of Sport and Physical Education of Sfax, Sfax (Tunisia), and the Higher Institute of Sport and Physical Education of Kef, Kef (Tunisia). All participants to the present study provided written, informed consent. Teachers were extensively informed about the purposes and procedure of the study, and were advised that the results would be made available to them upon completion of the study only in aggregate form, with no possibility to trace back to the single teacher's scores, thus ensuring anonymity and preserving the privacy of each participant.

## Author Contributions

NC, SG, FA, and NB conceived the experiment. NG and NB collected and analyzed data. NC, NG, SG, FA, and NB drafted the manuscript. NC, NG, SG, TR, JM, FA, and NB critically reviewed and approved the final version of the manuscript.

### Conflict of Interest

The authors declare that the research was conducted in the absence of any commercial or financial relationships that could be construed as a potential conflict of interest.
